# Methemoglobinemia in the Setting of Consumption of Poppers and Preservation Pellets

**DOI:** 10.7759/cureus.81768

**Published:** 2025-04-05

**Authors:** Nicholas D Luke, Ramasamy Muthuraman, Brianna Goldstein, Mehrdad Alaie

**Affiliations:** 1 Emergency Medicine, St. Barnabas Hospital Health System, New York City, USA

**Keywords:** acquired methemoglobinemia, acute respiratory failure with hypoxia, amyl nitrate, cyanosis, methylene blue

## Abstract

Methemoglobinemia can be either an acquired or congenital condition in which the state of the iron atom is altered pathologically, and it cannot adequately deliver oxygen molecules to peripheral tissues. This may be due to drug or environmental exposures, such as antibiotics and local anesthetics, or patients may have a congenital defect in specific hemoglobin proteins or enzymes. We present a patient who presented with peripheral cyanosis and respiratory distress without a clear initial cause. It was later identified that the patient had swallowed an entire bottle of ‘poppers’ with preservation pellets, which led to an acute state of methemoglobinemia. The patient was treated with methylene blue and placed on observation for airway management. With many recreational sources of potential causes of methemoglobinemia, including over-the-counter drugs and even vegetables, clinicians must take a thorough history to look for the root cause of the presenting symptomatology.

## Introduction

Methemoglobinemia is when a patient becomes hypoxic due to a diminished oxygen-carrying capacity of hemoglobin [1]. This condition can occur in the instance of drug use or exposure as an acquired condition. It may also present as a congenital process via autosomal recessive inheritance of abnormal cytochrome b5 reductase or autosomal dominant inheritance of mutations impacting several globin proteins known as hemoglobin M [1]. Methemoglobinemia can be life-threatening due to the hypoxia it causes from the modified hemoglobin. The modification of the hemoglobin leads to iron in the heme group in the Fe³⁺ state (which cannot bind oxygen), unlike the Fe²⁺ state of normal hemoglobin [2]. As the body becomes hypoxic, the remaining Fe²⁺ atoms will bind oxygen with greater affinity, thus leading to a leftward shift in the oxygen dissociation curve [2]. This process reduces tissue perfusion even further. Several drugs that may cause acquired methemoglobinemia include nitrites, benzocaine, and dapsone. Recreational use of ‘poppers,’ which contain nitrites, can lead to methemoglobinemia. The recreational use of ‘poppers’ leads to feelings of euphoria and can serve as a potent vasodilator before intimacy. Patients may present with a variety of symptoms, including dyspnea, fatigue, headache, seizures, and death. We present a patient who came to the emergency room with hypoxia, nausea and vomiting, and peripheral cyanosis, who was eventually admitted after drinking an entire bottle of a substance that is an analog of amyl nitrite with pellets for prolonging the substance’s potency. 

## Case presentation

**Figure 1:**
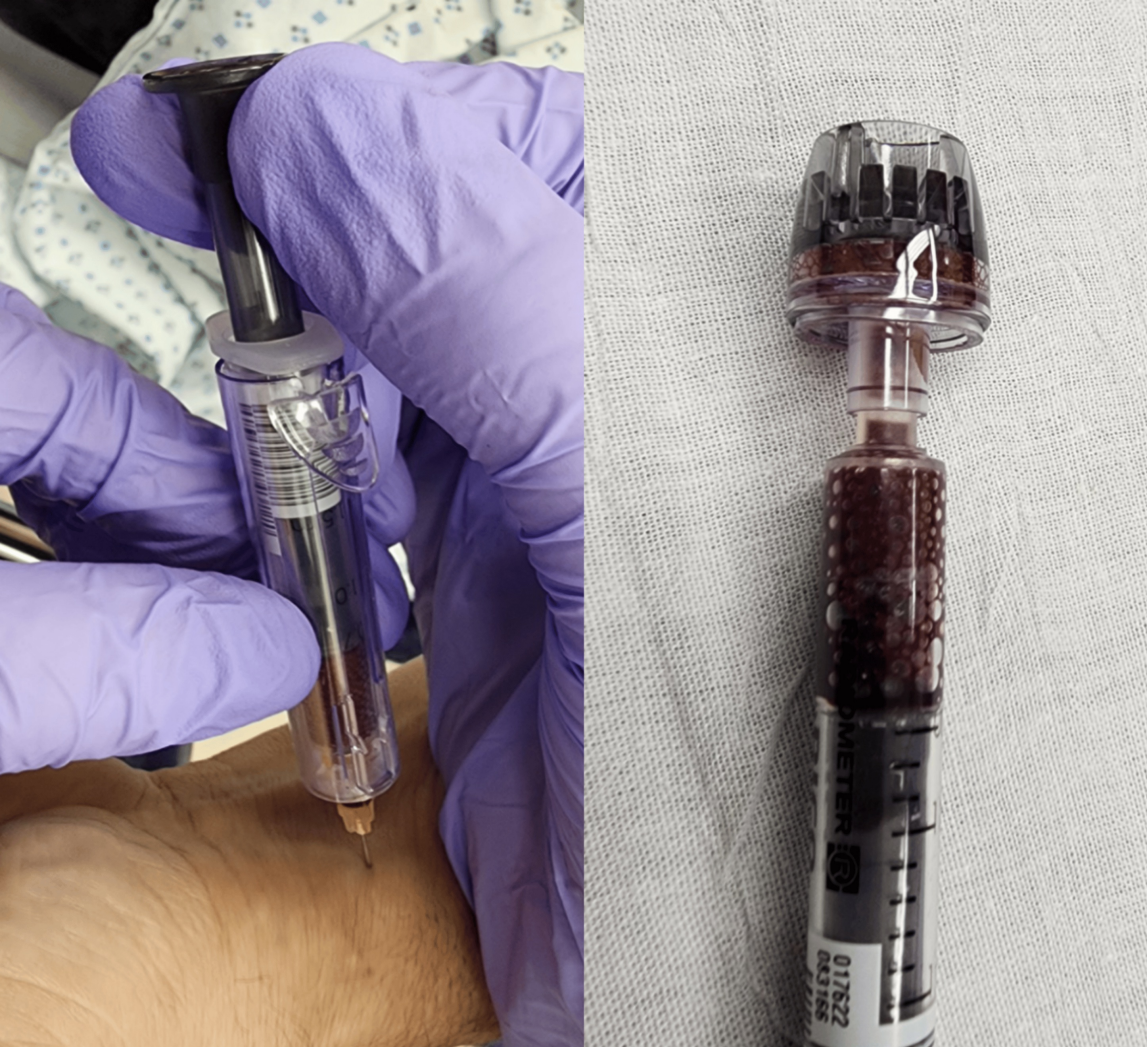
An arterial blood gas is drawn from the patient. The color of the blood is noticeably darker than expected from an arterial draw; this further reinforces the diagnosis of methemoglobinemia.

A 50-year-old male patient with a past medical history of type 2 diabetes, hypertension, hyperlipidemia, and human immunodeficiency virus (HIV) who was compliant with his HIV medications presented with headache, nausea, vomiting, and blue fingers as per the patient. Initially, he denied any drug use. He denied recent travel, diet changes, chest pain, shortness of breath, leg swelling, and surgical history. The patient’s initial vital signs were as follows: a heart rate of 120 beats per minute, oxygen saturation of 87% on room air, a respiration rate of 22 breaths per minute, and a blood pressure of 155/93 mmHg. His oxygen saturation increased to 89% when placed on a nonrebreather mask. The patient was in mild respiratory distress but was protecting his airway and was noted to have peripheral cyanosis in all of his fingers on the exam. The remainder of the physical exam was unremarkable. The patient was given medications for symptomatic control of his nausea and vomiting. Blood work was started, and the color of the blood was noted to be darker than expected from an arterial blood gas collection (Figure 1). A chest X-ray was performed, and it was unremarkable, with no signs of pneumonia, pleural effusion, or pneumothorax. The blood results were significant for a white blood cell count of 18.8 10*3/uL; the initial arterial blood gas was remarkable for an oxygen saturation of 92% and a partial pressure of oxygen of 342 mmHg. The remainder of the blood work was within normal limits, including blood glucose (194 mg/dL), thyroid-stimulating hormone (1.2 mIU/L), magnesium (2.1 mg/dL), phosphate (3.6 mmol/L), bicarbonate (23.2 mEq/L), lactic acid (1.4 mmol/L), blood pH (7.36 pH units), anion gap (15 mEq/L), and the partial pressure of carbon dioxide on the arterial blood gas (43.6 mmHg).

At this point, the patient was not in respiratory distress but was still saturating at 89% on the nonrebreather mask. There was a discussion about starting the patient on non-invasive ventilation. Since the patient was nauseous and did not have an altered mental status, the nonrebreather mask was kept in order to minimize the risk of aspiration. After multiple attempts at interviewing the patient and establishing trust, he eventually stated that he was about to get intimate with his significant other but had drunk an entire bottle of poppers before sex. The symptoms occurred about half an hour after he drank the whole bottle, and then he presented to the emergency room within the next half hour. The volume of the bottle was nine milliliters. The bottle also had pellets inside to help preserve the potency of the isobutyl and cyclopentyl nitrite (the ingested popper), an analog of amyl nitrite. Poison control was contacted at this point, and they recommended obtaining a methemoglobin level and starting methylene blue at 2 mg/kg over five minutes if the level is elevated. Then, the patient will be re-evaluated, and the methylene blue may be given again (at 2 mg/kg) after 30 to 60 minutes based on the patient's clinical presentation. The initial methemoglobin level was 36.7%. The target level for clinical resolution is less than 5%. The patient was then observed for five hours after receiving an initial dose of methylene blue of 180 milligrams. After the observation period, the repeat arterial blood gas showed an oxygen saturation of 98%, and the remaining blood gas values were all within normal limits. The repeat methemoglobin level was 1.5%, and the patient showed no respiratory distress on room air. The patient was subsequently discharged with return precautions at that point.

## Discussion

Methemoglobinemia is a potentially life-threatening disorder in which hemoglobin cannot adequately deliver oxygen to organs. The pathophysiologic process is due to the modification of the iron atom, which is transformed from a divalent to a trivalent form (from ferrous to ferric) [3, 4]. The symptomatology of methemoglobinemia is a direct result of inadequate oxygenation that causes a left shift in the oxygen-hemoglobin dissociation curve. Patients will present with hypoxia, shortness of breath, altered mental status, and peripheral cyanosis. Methemoglobinemia can be classified as a functional anemia since there are normal levels of iron and hemoglobin [3]. It may be inherited as a congenital effect in the CYB5R3 gene or hemoglobin M, or it can be acquired due to exposure to drugs and environmental hazards [3, 4].

Whether the methemoglobinemia is acquired or congenital, the first-line treatment will be methylene blue. This drug reverses the trivalent iron atom back into a divalent atom, thus permitting the normal function of carrying oxygen to peripheral tissues. The typical dose is one to two milligrams per kilogram of body weight, given intravenously and slowly over a few minutes [5]. The maximum dose that can be given is seven milligrams per kilogram. The drug is metabolized by the liver and excreted by the kidneys, so adjustments may be needed when dosing patients with renal or hepatic dysfunction at baseline [5].

Methemoglobinemia is known to occur in the setting of certain drugs, such as benzocaine, nitrates, and dapsone [6]. It can also happen in environmental exposure, such as fertilizers and herbicides [3]. Our patient had acquired methemoglobinemia in the setting of consuming an entire bottle of ‘poppers’ with the preservation pellets. The usual time of onset of poppers is within seconds if inhaled. Our presenting patient drank the whole bottle and subjectively stated that he experienced the adverse effects within the next half hour or so. When inhaled, poppers can last approximately a few minutes. The underlying cause of the methemoglobinemia in our presenting patient is the isobutyl and cyclopentyl nitrite found in the substance, an analog of amyl nitrite. Amyl nitrite can induce methemoglobinemia via oxidation of iron within hemoglobin, which causes an irreversible binding to oxygen molecules. Cannata et al. presented a case of methemoglobinemia in which a patient consumed a homemade vegetable puree [7]. Cannata et al. ruled out other possible causes of methemoglobinemia based on the history taken [7]. Cannata et al. discussed how cyanosis in their patient occurred after the puree's consumption, and vegetables contain nitrates that are converted into nitrites via gut microbiota [7]. The methemoglobin produced via vegetables is usually insufficient to cause symptoms unless the diet is primarily vegetables and the patient's age is less than six months old, as this patient population has an immature methemoglobin reductase system [7]. Cannata et al.’s patient was about 10 months old, so it may be possible that the vegetables used in the puree were stored for a prolonged period, which can concentrate the nitrate content [7]. Cannata et al.’s case portrays how methemoglobinemia can occur in settings that may not always be at the forefront of a clinician's differential. Thus, being aware of such scenarios when working up a patient presenting with methemoglobinemia is helpful. This can also be seen with congenital methemoglobinemia. In a report written by Paudel et al., a patient was diagnosed with congenital methemoglobinemia at the age of 33, even though she had multiple episodes of cyanosis and hypoxia throughout her life [8]. Although Paudel's patient did not exhibit severe symptoms, she did pass it on to her children, and this warrants genetic evaluation of immediate family members to analyze the etiology [8].

Methemoglobinemia can sometimes present as polycythemia vera due to the cyanosis and persistent elevation of red blood cells [9]. Soliman et al. discuss a case of congenital methemoglobinemia that became apparent due to an elevated red blood cell count, with typical symptoms of hypoxia secondary to methemoglobinemia [9]. Our presenting patient did not present with polycythemia due to the acute state of exposure to methemoglobinemia-inducing chemicals. Still, congenital cases are more likely to present with abnormal values due to chronicity. The range of symptoms of methemoglobinemia can vary in severity from mild to severe; however, it should be considered in patients with unexplained cyanosis and hypoxia at any age bracket. Another example of misdiagnosed methemoglobinemia, Tasci et al. described a case in which a patient was diagnosed with asthma for five years before their diagnosis of methemoglobinemia was discovered [10]. Tasci et al.'s patient was hypoxic, and there was a concern that the reason for their visit to the clinic was more than just an asthma exacerbation [10]. They ruled out pulmonary emboli, whether they may be acute or chronic, and the patient was still hypoxic [10]. The patient also had a sibling who had similar complaints and ultimately passed away due to undiagnosed congenital methemoglobinemia [10]. Once the patient's diagnosis was properly discovered as methemoglobinemia, the patient was treated promptly, and their hypoxia was resolved [10]. Although it may not always be at the forefront of a clinician's differential diagnosis, it should be considered in persistent hypoxia. Especially when the management of methemoglobinemia is relatively straightforward, the patient may avoid fatal outcomes as a result of a swift diagnosis. 

## Conclusions

Methemoglobinemia is a potentially life-threatening condition that leads to hypoxia secondary to modification of the iron atom found within hemoglobin. The diagnosis can be obtained by examining the results of a physical exam and measuring methemoglobin levels. The treatment involves the utilization of methylene blue, which will reverse the iron atom from a ferric to a ferrous state. It is pivotal to make the patient feel comfortable as emergency physicians so they feel at ease when providing history, enabling one to target the diagnosis. Without building rapport, this case would have been more challenging to analyze for the underlying cause of the presentation.
